# The role of wood anatomical traits in the coexistence of oak species along an environmental gradient

**DOI:** 10.1093/aobpla/plab066

**Published:** 2021-10-18

**Authors:** Maribel Arenas-Navarro, Ken Oyama, Felipe García-Oliva, Andrés Torres-Miranda, Enrique G de la Riva, Teresa Terrazas

**Affiliations:** 1 Posgrado en Ciencias Biológicas, Unidad de Posgrado, Universidad Nacional Autónoma de México, Ciudad Universitaria, Coyoacán CDMX CP 04510, México; 2 Escuela Nacional de Estudios Superiores (ENES) Unidad Morelia, Universidad Nacional Autónoma de México, Antigua Carretera a Pátzcuaro 8701, Morelia, Michoacán CP 58190, México; 3 Instituto de Investigaciones en Ecosistemas y Sustentabilidad, Universidad Nacional Autónoma de México, Antigua Carretera a Pátzcuaro 8701, Morelia, Michoacán CP 58190, México; 4 Department of Ecology, Brandenburg University of Technology, 03046 Cottbus, Germany; 5 Instituto de Biología, Universidad Nacional Autónoma de México, Ciudad Universitaria, Coyoacán CDMX CP 04510, México

**Keywords:** Aridity index, fibre traits, phenotypic plasticity, *Quercus*, relative hydraulic conductivity

## Abstract

Oaks (*Quercus*) are a dominant woody plant genus in the northern hemisphere, which occupy a wide range of habitats and are ecologically diverse. We analysed the wood anatomical traits, the variables derived and the relative hydraulic conductivity of 21 oak species to identify their performance according to abiotic factors, leaf phenological patterns and phylogenetic restrictions by analysing the interspecific variation along an environmental gradient. First, we determine the causes of anatomical trait variation in the oaks, analysing the functional trade-offs related to distribution along the environmental gradient. We measure the phenotypic plasticity of the anatomical traits to determine the role of environment and geographic distance in the range of phenotypic plasticity. Second, we examined if oaks co-occurred along the environmental gradient. Then we analysed if wood anatomical traits reflect differences among their phylogenetic section, leaf habit and a phylogenetic section/leaf habit category. Last, we tested the phylogenetic signal. Our results showed that vessel diameter, vessel frequency, wood density and relative hydraulic conductivity are the main axes of trait variation in the species analysed among leaf habit categories. The aridity index and seasonal precipitation drive the variation in the analysed traits. Higher environmental distance resulted in a higher relative distance plasticity index among traits. Co-occurrence of oak species with different leaf habits and phylogenetic trajectories may promote complementary resource acquisition. The phylogenetic signal in the oak species studied was low, which implies labile wood traits.

## Teaser

Wood anatomical traits reflect the adaptations along an aridity gradient in oak species. We found that high temperatures combined with periodic water deficits lead to narrower vessels, high-density wood occupied by smaller fibres. On the contrary, on humid sites, tall oaks invest in widest vessels to conduct water with great efficiency. Oak species in the study site showed an adaptive response of wood traits to climate, but there is also evidence of a low phylogenetic signal. Co-occurrence of oak species with different leaf habits and phylogenetic trajectories may promote complementary resource acquisition.

## Introduction

The ability of plants to modify their functional traits has been suggested to be associated with their distribution patterns and survival success (Díaz *et al.* 2016; Cosme *et al.* 2017). Species distributions result from trait composition through environmental filters, as water availability (Cosme *et al.* 2017). Water is essential for the growth and maintenance of woody plants; in angiosperms, water transport is conducted through vessels (and nutrients transport), but other cell types are also involved in the distribution of water as fibres (mechanical support) and parenchyma (metabolite transport and water storage) (Zanne *et al.* 2010; Ziemińska *et al.* 2015). Angiosperms vary significantly in the fraction of these cells observed in cross-sections of the secondary xylem and the spatial organization (Morris *et al.* 2018). Wood cells do not operate in isolation from one another and are anatomically and functionally integrated; the coupling of vessels and other cells varies across species and can be modified by climate and mechanical stability (Ziemińska *et al.* 2015; Morris *et al.* 2018).

The variation among wood traits has allowed identification of several trade-offs and includes the following: changes in the fraction of sapwood occupied by vessel lumina (open conduit spaces), between vessel diameter (VD) and vessel frequency (VF); the fraction of fibre wall (F_W_) and fibre lumen (F_L_) and the parenchyma-fibre fraction (Chave *et al.* 2009; Zanne *et al.* 2010; Ziemińska *et al.* 2015). These adjustments are not necessarily mutually exclusive, and plants may combine these traits to acquire and store water (Gratani 2014; Ziemińska *et al.* 2015).

In dry environments, plants invest in traits that confer hydraulic resistance to periodic water deficits without suffering significant cavitation or hydraulic failure (Baas *et al.* 2004; Sperry *et al.* 2008). For example, VD is reduced and compensated by increasing the number of vessels in the sapwood area to minimize the risk of embolism. Frequently, narrower vessels are immersed in a matrix of high-density wood due to the higher proportion of xylem occupied by fibres and thicker conduit walls relative to the lumen area, which increases the resistance to implosion (Hacke *et al.* 2001; Jacobsen *et al.* 2007; Fortunel *et al.* 2014). In contrast, in humid environments plant species invest in traits that conduct water with great efficiency. Xylem hydraulic efficiency can be achieved by investing in large and wide vessels, reducing water flow resistance and increasing conductivity (Hacke *et al.* 2006; Fan *et al.* 2011). Therefore, plants in environments with water availability exhibit a combination of wide and large vessels and low-density wood, maximizing growth and conferring a competitive advantage in the acquisition of light (Cosme *et al.* 2017; Z. Liu *et al.* 2019).

In regions where water resource availability is heterogeneous, niche partitioning facilitates the spatial segregation of plant species and promotes high diversity (Bartelheimer *et al.* 2010; Arenas-Navarro *et al.* 2020a). The spatial and temporal variation of water availability is a critical feature that selects trees’ contrasting leaf and wood traits and may explain plant distribution patterns (Apgaua *et al.* 2015; Cosme *et al.* 2017). Variation in wood anatomical traits along environmental gradients represents adaptive structural solutions to achieve an optimal balance among the competing needs of support, storage and transport (Chave *et al.* 2006; Swenson and Enquist 2007; Martínez-Cabrera *et al.* 2009). Interspecific differences among clades in wood anatomical and hydraulic traits along environmental gradients or contrasting sites reflect the differences in the way plants adapt or adjust their traits to environmental variability (Aguilar-Romero *et al.* 2017; Nabais *et al.* 2018; Rosas *et al.* 2019; Skelton *et al.* 2021). However, when phylogenetically conserved traits are correlated with specific environmental conditions, close relatives should respond similarly (Chave *et al.* 2006; Swenson and Enquist 2007), on the contrary, the lack of phylogenetic signal could imply evolutionary lability (Silvertown *et al.* 2006a). Different phylogenetic conservative traits have been identified indicating similar responses to environmental changes (Wiens *et al.* 2010). However, contrasting life forms, height and relative growth rates have also been detected for closely related species within a genus (Cavender-Bares *et al.* 2004; Liu *et al.* 2015).

Oaks (*Quercus*, Fagaceae) are woody plants that occupy diverse ecosystems ranging from tropical dry seasonal forests to cloud forests (Nixon 1993; Valencia-Á. 2004). The American oak lineages mainly comprise species of the *Lobatae* (red oaks) and *Quercus* (white oaks) sections, which represent a high proportion of the above-ground live biomass and biodiversity in Mexican forests (Valencia-Á. 2004; Cavender-Bares 2019). Oaks underwent an extraordinary diversification in Mexico along the different mountain chains (Nixon 1993; Hipp *et al.* 2018), which resulted in several endemism centres (Torres-Miranda *et al.* 2013). Previously, we found that oak species in the region of Serranias Meridionales de Jalisco (SMJal) showed that the functional space (measured with leaf and stem traits) was linked to niche segregation (Arenas-Navarro *et al.* 2020a). The results suggest that the earliest mechanism of species segregation was related to habitat suitability and that the stem trade-off reflects differences between *Quercus* and *Lobatae* sections. Hence, it is important to incorporate phylogenetic relatedness, which is essential in comparative studies focusing on a single trait’s evolutionary patterns or in pairs of traits. In this study, our main goal was to determine associations between the wood anatomical traits in oak species, analysing if the phylogenetic relationship and the leaf habit influence the variation of the traits and the coexistence of the species along the environmental gradient. First, we determine what are the causes of anatomical trait variation in the oaks, analysing the functional trade-offs related to distribution along the environmental gradient in the SMJal. Also, we measure the phenotypic plasticity of the anatomical traits to determine the role of environment and geographic distance in the range of phenotypic plasticity. Second, we analysed the local competition; for this, we analysed if oaks co-occurred along the environmental gradient. We then analysed if wood anatomical traits reflect differences among their phylogenetic section, leaf habit and among phylogenetic section/leaf habit category. Last, we tested the phylogenetic relatedness analysing if phylogenetically conserved traits exist among the oak species analysed. We hypothesize that lowland oak species in areas with higher temperature and marked rainfall seasonality converge on drought-resistant functional traits and segregate between species that belong to the same section; in contrast to highlands in humid areas with less temperature, the traits will tend to diverge between species to avoid local competition.

## Materials and Methods

### Site description

The study site was conducted in the floristic province of SMJal, in west Mexico which comprises the ‘El Tuito-El Cuale-Talpa de Allende’ Sierras (20°20.885′–20°9.008′N, and 105°19.162′–104°40.106′W). This mountain complex is located within Cabo Corrientes and Talpa de Allende counties in Jalisco state, Mexico; in the boundary limits of the biogeographic provinces of the Sierra Madre del Sur and the Trans-Mexican Volcanic Belt with an altitudinal gradient that ranges from 650 to 2738 m **[see****Supporting Information—Fig. S1****]**. The annual mean temperature ranges from 28.5 to 30.6 °C and the annual mean precipitation from 1500 to 1800 mm, with 80 % of the rainfall concentrated between June and October (SMN 2017). The main vegetation types are tropical deciduous forests at low elevations, oak, pine-oak and fir forests at high elevations and montane cloud forests in glens (Arenas-Navarro *et al.* 2020b).

### Oak’s species and leaf habit

Previously 33 rectangular 0.1 ha plots (50 × 20 m) were established to conduct vegetation sampling along the elevational gradient (Arenas-Navarro *et al.* 2020b). In each plot, all trees with diameter at breast height (DBH) > 15 were registered. We recorded 21 oak species in the plots (**see****Supporting Information—Table S1** for species names); at each plot, we selected five adult individuals of each oak species present, sampling 275 oak individuals in total to measure wood functional traits. For each tree, we recorded height (m) and DBH (cm). The category of leaf habit was based on monthly canopy foliage duration records for 6–10 individuals per species over 1 year (from February 2017 to January 2018) along with their altitudinal distribution. We counted the number of leaves present in three sun-exposed terminal twigs and quantified the percentage of the total canopy. The period of canopy foliation was calculated as the number of days per year that a tree maintained 50 % or greater of its foliage relative to maximum foliage. Species were coded as deciduous when leaves drop seasonally and are absent for a significant portion of the year; brevideciduous when leaves are present less than year-round or with a brief period of leaflessness, evergreen with a canopy present year-round (Arenas-Navarro *et al.* 2020a).

### Wood trait measurements

For wood traits, we removed a wood slide less than 5 cm in width at ~1.3 m trunk height with a saw in each tree, avoiding the reaction wood because of the steep slope in some plots. Samples were stored in sealed plastic bags cooled until measurements in the laboratory. For this slide, we removed the bark, and wood from sapwood was divided into woodblock sections. From one woodblock, we determined wood density by the water-displacement method (Pérez-Harguindeguy *et al.* 2013). The saturated wood sample was immersed into a beaker of water loaded on an electronic balance. The wood sample was pressed below the water surface with an insect pin and the volume of the wood was obtained as the mass of the displaced water. After volume measurement, the wood sample was dried in the oven at 101 °C for 72 h, until a constant weight is obtained. The remaining portions of the wood samples were fixed in glycerine–ethanol–water (1:1:1). Lastly, transverse and longitudinal sections were cut 20 µm thick with a sliding microtome (Leica 2000 R, Westlar, Germany). Sections were double stained with safranin and fast green (Ruzin 1999) and mounted with synthetic resin. For VD, the tangential vessel diameter of 50 vessels per individual was counted (Table 1). Vessel frequency was calculated in 1 mm^2^ considering all the vessels within two of the widest rays in 25 optical microscopic fields. Fibres were separated into three fractions: fibre wall (F_W_), fibre lumen (F_L_) and fibre total diameter (F_D_), quantifying 25 fibres per individual. Anatomical traits were measured using an image analysis program (Image-Pro v.7.1 connected to an Olympus BX50 light microscope). In addition, four variables derived from the anatomical traits were calculated for each individual as vessel composition index (S; Zanne *et al.* 2010), vessel lumen fraction (F; Zanne *et al.* 2010), vulnerability index (VI; Carlquist 1977) and relative hydraulic conductivity (RC; Carlquist 1988).

**Table 1.** Wood anatomical and hydraulic traits measured in oak species.

Anatomical traits	Unit	Description/formula
VD	μm	Vessel diameter (average equivalent 50 circle diameter per individual)
VF	per mm^2^	Vessel frequency, i.e. number of vessels per mm^2^
VA	μm^2^	Vessel area (average equivalent 50 circle area per individual)
WD	g cm^−3^	Wood density
F_D_	μm	Fibre total diameter (average 25 per individual). F_D_ = F_L_ + 2(F_W_)
F_L_	μm	Fibre lumen diameter (average 25 per individual)
F_W_	μm	Fibre wall thickness (average 25 per individual)
Variables		
S	–	Vessel composition index; S = VA/VF
F	–	Vessel lumen fraction; F = VF * VA
VI	–	Vulnerability index = VD/VF
RC	–	Relative hydraulic conductivity: RC = *r*^4^VF (*r* is the radius of VD)

### Relative distance plasticity index

The relative distance plasticity index (RDPI) was calculated to test for overall plasticity of species according to Valladares *et al.* (2006). Relative distance plasticity index calculates the distance of one trait between two individuals: individual *j* of a particular species × growing under condition *i* with a second individual *j′* of the same species × growing under another condition *i′*. The sum of one pair is then divided by the total number of compared pairs or distances *n*. Relative distance plasticity index ranged from 0 (no plasticity) to 1 (maximal plasticity) (Equation (1)).


$$\begin{array}{l} RDPI=\sum (d_{ij}\to i^{\prime }j^{\prime }/(x_{i^{\prime }j^{\prime }}+x_{ij}))/n \end{array}$$ (1)

Equation (1)—Equation for the RDPI.

Relative distance plasticity index has the advantage of not assuming any particular distribution of the data and significantly increasing the power of the statistical analyses (Valladares *et al.* 2006). Relative distance plasticity index was calculated for 19 species with ‘Plasticity’ R package (Ameztegui 2017), excluding *Quercus martinezii* and *Q. uxoris* because they were collected each in a single plot.

### Environmental variables

The environmental variables for the study site were obtained using the climatic surfaces reported by Cuervo-Robayo *et al.* (2014) at 60 m^2^ resolution (Arenas-Navarro *et al.* 2020b). Nineteen environmental variables were extracted for each plot. Aridity index (AI) proposed by the United Nations Environment Programme (UNEP) was calculated (Allen 2006). Aridity index is a numerical indicator of the degree of dryness of the climate at a given location; values close to zero reflect hyper aridity, and AI ≥ 0.65 is classified as humid (UNEP 1997). Potential evapotranspiration was calculated with the Hargreaves and Samani method (Hargreaves and Samani 1985). To avoid multicollinearity among the environmental variables, we selected variables with less correlation, we reject variables that result in variance inflation factors (VIFs) > 10 and we additionally check collinearity among the selected environmental variables by a principal components analysis (PCA; Quinn and Keough 2002). The climatic variables selected were AI, mean temperature of the driest quarter (mtdq), precipitation of the warmest quarter (pwaq), precipitation of the wettest quarter (pwq) and precipitation seasonality (ps).

### Data analyses

#### Correlations among traits.

The strengths of relationships among traits were determined using Pearson’s correlations and phylogenetically independent contrasts (PICs) to account for non-independence of data due to phylogenetic relationships (Felsenstein 1985; Martínez-Cabrera *et al.* 2011). All traits were log_10_-transformed to improve the normality criteria. The contrasts were constructed by analysing each species as an independent point using the ‘pic’ function of the ‘ape’ package ver. 5.5 in R (Paradis and Schliep 2019). The relationships between pairs were made with linear regressions, adjusting the ordinate to the origin to zero. In addition, two PCA analyses (with traits and PICs) were conducted to determine how oak trees occupy different regions of multivariate trait space. To avoid redundancy among traits in the PCA, VA and S were discarded in the traits PCA.

#### Plasticity and environmental and geographic distance.

We obtained the average of the RDPI by each species for the wood traits analysed from one plot to another plot and constructed a distance matrix per phylogenetic section. We used multiple regression on distance matrices (MRM) (Legendre and Legendre 1998; Lichstein 2007) to determine the extent to which geographic distance and environmental distance influence the RDPI. Multiple regression on distance matrices performs a multiple regression analysis between two or more distance matrices, using permutations to determine the significance of the coefficients of determination (Legendre and Legendre 1998; Lichstein 2007). Multiple regression on distance matrices was conducted using RDPI distances as response and geographical and environmental distance matrices as predictors. The geographical distance matrix was calculated with the geographical distances between each pair of plots, using their geographical coordinates and Euclidean distances. To construct the environmental distance matrix, we standardized the different units of the different environmental variables and used these values to build up a distance matrix based on Euclidean distances by pair of plots. Multiple regression on distance matrices analyses were conducted using 5000 random permutations of the rows and columns of the dependent matrix. Multiple regression on distance matrices was performed in ‘*ecodist*’ ver. 2.0.1 (Goslee and Urban 2007) in R. Relative distance plasticity index values were *arcsin-square* root transformed prior to analysis (Grassein *et al.* 2010). Analyses were calculated in R software ver. 3.6.3 (R Core Team 2020).

#### Species co-occurrence.

The species co-occurrence analysis was calculated with pairwise associations between 21 oak species across 33 plots according to the probability model of species co-occurrence (Veech 2013; Arita 2016; Griffith *et al.* 2016). The model calculates the expected frequency of co-occurrence between each species pair based on the distribution of one species independent of the second one. Then compares the expected frequency to the observed frequency and returns the probability that a lower or higher value of co-occurrence could have been obtained by chance. Finally, the probabilities are interpreted as *P-*values, and the model classifies species pairs as significant positive, negative or random associations based upon an alpha threshold of 0.05 (Veech 2013; Griffith *et al.* 2016). We used the ‘cooccur’ ver. 1.3 package in R (Griffith *et al.* 2016).

#### Test for phylogenetic signal.

**Figure 1.**
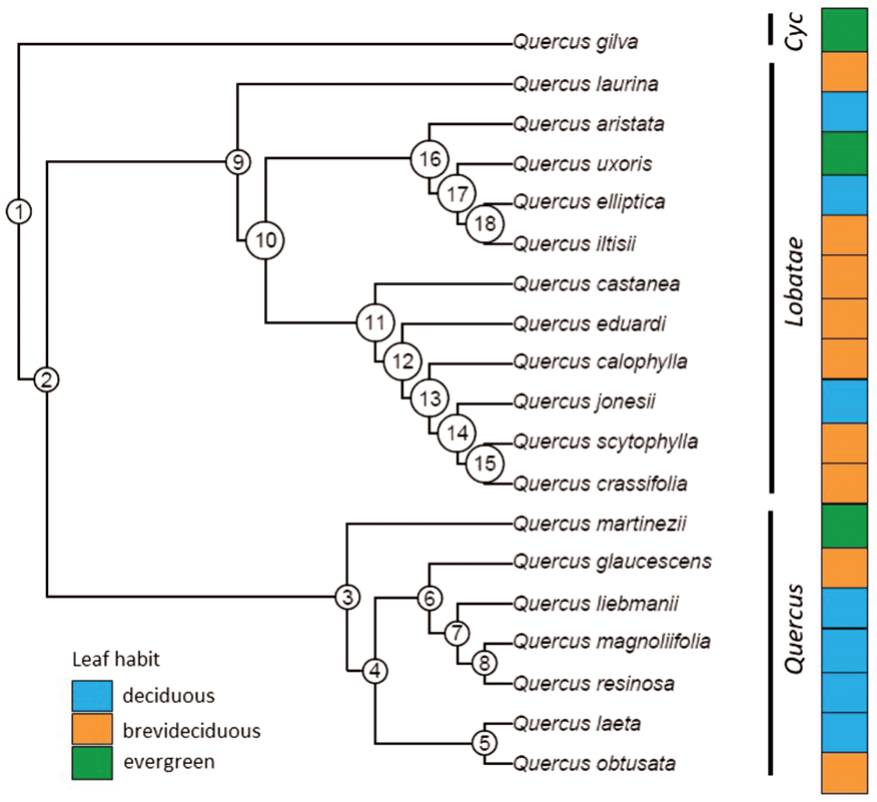
Phylogeny of 19 oak species. Species from *Quercus* and *Lobatae* sections are the species recorded for this study, indicated in the black bars. The panel of the right indicates leaf habit. Numbers indicate intern nodes of the phylogeny.

Patterns of trait variation among related species sometimes reflect phylogenetic relationships, such that more closely related taxa are the species that share similar trait values (Blomberg *et al.* 2003). A phylogenetic tree was pruned to include the species from the present study, based on the ‘singletons tree’ of the Global Oak Phylogeny project (Hipp *et al.* 2020; Fig. 1). *Quercus cualensis*, *Q. tuitensis* and *Q. mexiae* are missing from this data set and were excluded from the tree and further phylogenetic analyses. *Quercus gilva*, a species from China from section *Cyclobalanopsis*, was incorporated as an extern group (Hwang 1962; Xu *et al.* 2019). We investigated phylogenetic signals for all traits using Blomberg’s *K*. *K* measures the extent to which a trait displays phylogenetic signal, where *K* = 0 indicates no phylogenetic signal, *K* = 1 suggests that the trait distribution perfectly conforms to Brownian motion and *K* > 1 indicates stronger similarities among closely related species than expected under Brownian motion. We did this analysis with the ‘picante’ ver. 1.8.2 package in R (Kembel *et al.* 2010).

We analysed the effect of phylogenetic history using a phylogenetic ANOVA to test the differences among phylogenetic sections (red and white oaks), among leaf habit categories and among a phylogenetic section/leaf habit category. The categories among section/leaf habit only were considered for the deciduous and brevideciduous species due to the low number of evergreen species. We used the function ‘*phylANOVA*’ for the package ‘*phytools*’ (Revell 2012) in R with 1000 simulations and Holm’s method for *P*-value adjustment and later we made *post hoc* analyses if we found differences among groups.

To examine the effect of environmental variables on traits while accounting for phylogenetic relationships we used phylogenetic generalized least squares (pgls). For each trait, we selected the best model among the five environmental variables with the lowest Akaike information criterion. We used the function ‘*pgls*’ for the package caper v. 0.2 (Orme *et al.* 2018). Because phylogenetic signal is sensitive to small sample sizes (<20; Münkemüller *et al.* 2012), we also used ordinary least square models (ols), which ignored phylogenetic relatedness and we present both results.

## Results

### Correlations among traits

Pearson’s correlations showed a high correlation for anatomical traits between vessel diameter and vessel area (*R* = 0.99); thus, we decided to discard vessel area from further analyses. Also, vessel diameter showed a high correlation with the anatomical variables (*R* > 0.67) (Table 2). Vessel frequency is negatively correlated with vessel diameter (*R* = −0.51), vulnerability index (*R* = −0.89) and vessel composition index (*R* = −0.83). Fibre total diameter is correlated with fibre wall thickness (*R* > 0.71), but it is not significantly correlated with the fibre lumen diameter (*R* > 0.39). Vulnerability index is positive correlated with relative hydraulic conductivity (*R* = 0.56) and vessel composition index (S; *R* = 0.97); RC is positive correlated with vessel lumen fraction (*R* = 0.86) and S (*R* = 0.69). For last tree height was positively correlated with vessel diameter (*R* = 0.69), fibre lumen diameter (*R* = 0.6), vulnerability index (*R* = 0.63), RC (*R* = 0.55) and vessel composition index (*R* = 0.64). The PICs among traits yielded similar results to the Pearson’s correlations (Table 2). Some of the differences are the relationships between fibre total diameter and fibre lumen diameter (*R* = 0.28); fibre lumen diameter and wood density (*R* = 0.25).

**Table 2.** Correlation between pairs of traits. Pearson’s coefficients of correlations between pairs of traits. Pearson’s coefficients among species means are given below the diagonal, and correlations among phylogenetic independent contrasts (PICs) are given above it. Significant correlations are shown in bold (*P* < 0.05).

	VD	VF	VA	F_D_	F_L_	F_W_	WD	VI	RC	F	S	H
VD		**0.18**	**0.98**	−0.04	0.05	−0.05	0.04	**0.55**	**0.7**	**0.21**	**0.64**	**0.57**
VF	**−0.51**		0.15	−0.05	−0.04	−0.04	−0.05	**0.81**	−0.05	**0.2**	**0.71**	0.005
VA	**0.99**	**−0.51**		−0.04	0.07	−0.05	0.04	**0.52**	**0.76**	**0.24**	**0.63**	**0.58**
F_D_	0.15	0.04	0.15		**0.28**	**0.78**	−0.04	−0.05	−0.01	−0.03	−0.04	0.01
F_L_	0.28	−0.09	0.28	0.39		0.07	**0.25**	0.04	0.07	−0.02	0.05	**0.26**
F_W_	−0.07	0.03	−0.07	**0.71**	−0.22		−0.05	−0.05	−0.03	−0.04	−0.05	−0.01
WD	0.18	−0.16	0.18	−0.25	−0.42	−0.08		0.04	0.07	0.04	−0.03	0.06
VI	**0.81**	**−0.89**	**0.81**	0.01	0.22	−0.08	0.1		0.12	−0.03	**0.92**	**0.21**
RC	**0.92**	−0.19	**0.92**	0.25	0.26	0.01	0.17	**0.56**		0.61	0.2	0.45
F	**0.67**	0.29	**0.67**	0.21	0.23	−0.06	0.05	0.14	**0.86**		−0.05	0.19
S	**0.9**	**−0.83**	**0.9**	0.08	0.23	−0.06	0.2	**0.97**	**0.69**	0.29		0.31
H	**0.69**	−0.4	**0.69**	0.14	**0.6**	−0.18	−0.19	**0.63**	**0.55**	0.42	**0.64**	

**Figure 2.**
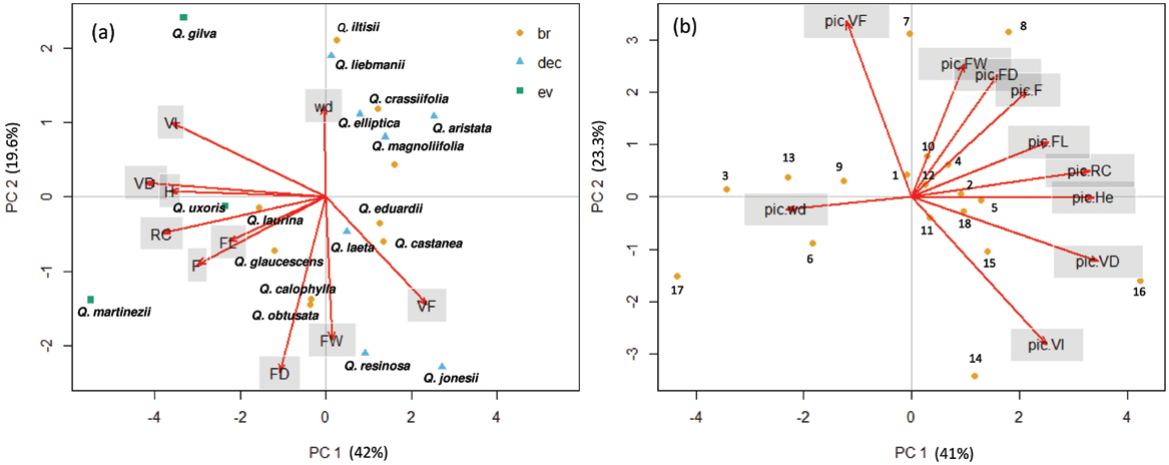
Principal components analysis (PCA). (A) PCA with species means for each trait VF (vessel frequency), VD (vessel diameter), WD (wood density), F_D_ (fibre total diameter), F_L_ (fibre lumen), F_W_ (fibre wall), vessel lumen fraction (F), vulnerability index (VI), relative hydraulic conductivity (RC) and tree height (H). Brevideciduous (br), deciduous (dec) and evergreen (ev). (B) PCA with the phylogenetic independent contrast (PIC) for traits. Numbers indicate intern nodes of the phylogeny (see Fig. 1).

**Figure 3.**
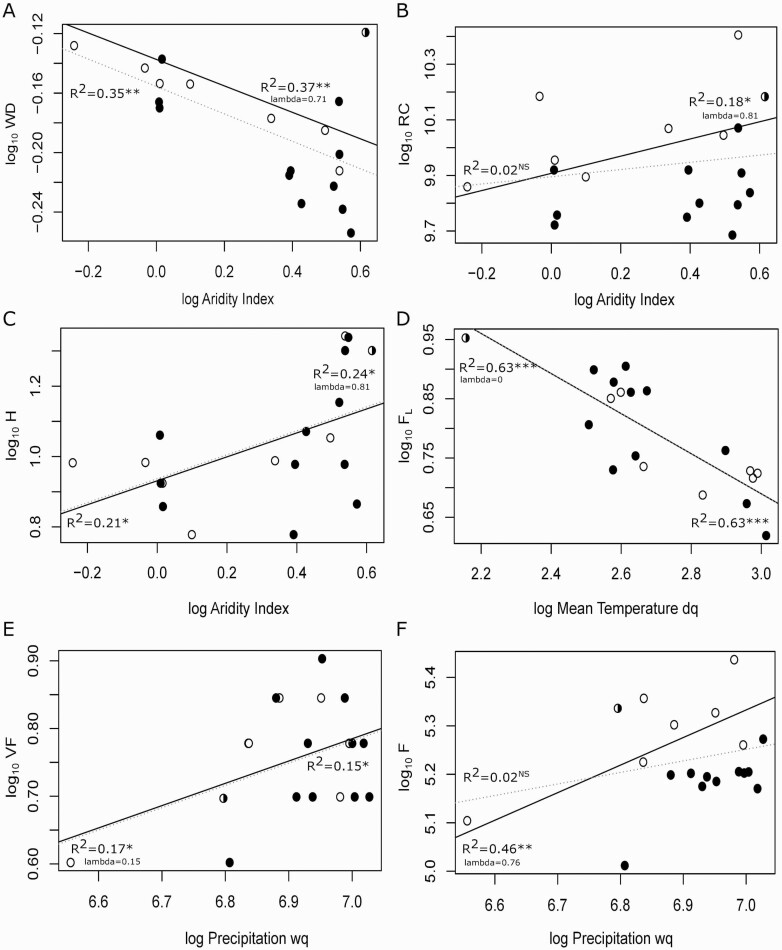
Relationships between anatomical traits and environmental variables. Regression bold lines are the phylogenetic generalized linear model (pgls), and regression grey dotted lines are the ordinary least square (ols) model. Significance levels are shown. NS = no significative; *P < 0.05; **P < 0.01; ***P < 0.001. Black circles are species from *Lobate* section; white circles are species from *Quercus* section and half black circle is *Q. gilva*. Acronyms: relative hydraulic conductivity (RC), tree height (H), wood density (WD), fibre lumen (F_L_), vessel frequency (VF) and vessel lumen fraction (F). Mean temperature of driest quarter (Mean Temperature dq), precipitation of wettest quarter (Precipitation wq).

The traits PCA showed that the three principal components explained 76.98 % of the total variance. The first axis (PC_1_) explained 42.02 % of the variance and was related to the trade-off between vessel diameter and frequency (Fig. 2A). The second axis (PC_2_) explained 19.58 % of the total variance and was explained by fibre total diameter and wood density, and the third axis (PC_3_) explained 15.38 % of the total variance and was explained by fibre lumen diameter and wood density **[see****Supporting Information—Table S2****]**. The second PCA performed with PICs reflected that the first three components of the PCA explained 82.68 % of the total variance (Fig. 3B). The first axis (PC_1_) explained 41.01 % of the variance related to the vessel diameter and wood density. The second axis (PC_2_) explained 23.34 % of the variance and was explained by the negative relationship between vessel frequency and vulnerability index. The third axis (PC_3_) explained 18.32 % of the variance and was explained by vessel lumen fraction and fibre wall thickness.

### Trait–environment relationship

The pgls analysis determined that vessel frequency and vessel lumen fraction were significantly affected by the precipitation of the wettest quarter (Fig. 3; **see****Supporting Information—Table S3**). The highest amount of precipitation of the wettest quarter leads to a higher vessel frequency and vessel lumen fraction. Fibre lumen diameter was significantly affected by the mean temperature of the driest quarter, sites with the highest temperatures possess narrower fibre lumen. Wood density, relative hydraulic conductivity and tree height were significantly affected by the aridity index (AI), in sites with lower AI developed higher wood densities, and higher plant height with higher RC are in most humid areas. Vulnerability index is significantly related with precipitation of warmest quarter, sites with lower precipitation in spring have higher vulnerability index values. Neither vessel density, fibre total diameter and wall thickness showed significant relationship with the environmental variables analysed by the pgls.

### Relative distance plasticity index

The oak species showed low RDPI values in the traits and variables analysed, which ranged from 0.009 to 0.43 **[see****Supporting Information—Fig. S2****]**. The species that showed the highest RDPI values were *Q. elliptica* (VD = 0.13; **see****Supporting Information—Fig. S2A**), *Q. castanea* (VF = 0.26) and *Q. aristata* for the fibre fractions (F_L_ = 0.33; F_W_ = 0.33). The species that showed the highest RDPI values in hydraulic variables were *Q. castanea* (VI = 0.30) and *Q. elliptica* (RC = 0.43; *F* = 0.23; **see****Supporting Information—Fig. S2B**). We performed a Student’s *t*-test among sections with the RDPI values and we found that red oaks species possess higher values of RDPI in vessel frequency (*t* = 3.313; df = 16.362; *P* = 0.004), vulnerability index (*t* = 3.313; df = 16.988; *P* = 0.004) and vessel lumen fraction (*t* = 3.220; df = 14.711; *P* = 0.005).

The MRM showed that environmental distance is a better predictor than geographical distance in both sections **[see****Supporting Information—Fig. S3****]**. For the red oaks the increase in RDPI for vessel diameter was greater, to a higher environmental distance (*R*^2^ = 0.58; *P* = 0.0002), while relative hydraulic conductivity showed the highest relationship (*R*^2^ = 0.61; *P* = 0.0002). For the white oaks, the greater relationship was for the RDPI for fibre wall thickness (*R*^2^ = 0.75; *P* = 0.0002) and vessel lumen fraction (*R*^2^ = 0.67; *P* = 0.0002).

### Species co-occurrence

**Figure 4.**
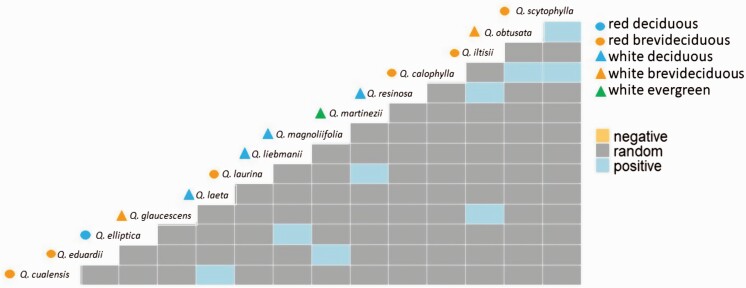
Oak species co-occurrence matrix. Heat map showing the positive and negative species associations determined by the probabilistic occurrence.

We analysed 231 species pairs, and the probabilistic modelling of species co-occurrence revealed zero negative associations, nine positive associations (3.89 %) and 222 random associations (96.11 %; Fig. 4). The positive species pairs were formed mostly among red and white oaks (eight species pairs) and one pair composed of two red oaks species. Of these pairs of species, three species pairs are formed by red brevideciduous and white deciduous (*Quercus cualensis*–*Q. laeta*, *Q. eduardii*–*Q. magnoliifolia* and *Q. iltisii*–*Q. resinosa*); three species pairs among red brevideciduous and white brevideciduous (*Q. iltisii*–*Q. glaucescens*, *Q. calophylla*–*Q. obtusata* and *Q. scytophylla*–*Q. obtusata*); one pair formed by red brevideciduous and white evergreen (*Q. laurina*–*Q. martinezii*); one pair formed by red deciduous and white deciduous (*Q. elliptica*–*Q. liebmanii*) and one pair formed by two red brevideciduous oaks (*Q. calophylla*–*Q. scytophylla*).

### Differences among phylogenetic section, leaf habit and among a phylogenetic section/leaf habit category

**Figure 5.**
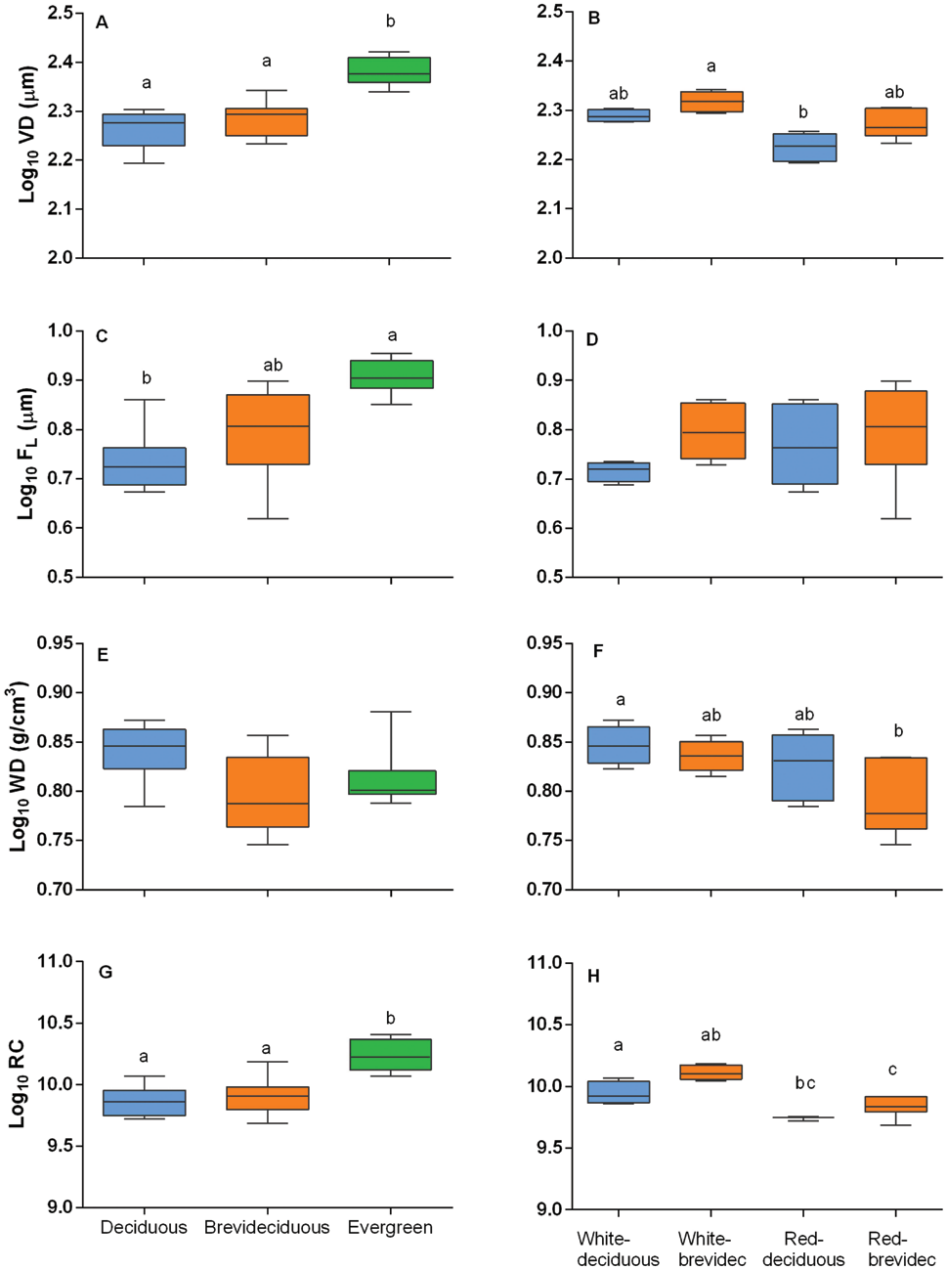
Boxplots among leaf habit and section/leaf habit. The section/leaf habit categories are for the deciduous and brevideciduous species. Different letters indicate *P* > 0.05 among groups with *post hoc* test. VD (vessel diameter), F_L_ (fibre lumen), WD (wood density) and RC (relative hydraulic conductivity).

The phylogenetic ANOVA among white and red oaks did not show significant differences among phylogenetic sections **[see****Supporting Information—Table S4****]**. Contrary, among the leaf habit categories, we found several significant differences. Vessel diameter and fibre lumen showed significant differences (*F* = 12.02; *P* = 0.001 and *F* = 4.8; *P* = 0.04, respectively) (Fig. 5). *Post hoc* analyses showed that evergreen species possess wider vessel diameter than deciduous (*t* = 4.4, *P* = 0.009) and brevideciduous species (*t* = 3.8; *P* = 0.014); also, evergreen oaks possess wider fibre lumen than deciduous oaks (*t* = 3.09; *P* = 0.03). Vulnerability index, relative hydraulic conductivity, vessel lumen fraction and vessel composition index showed significant differences among leaf habits category; *post hoc* analyses showed that evergreen species are significantly different from deciduous and brevideciduous species but not among deciduous and brevideciduous species **[see****Supporting Information—Table S4****]**. Finally, the tree height also showed differences among leaf habit; evergreen oaks species are taller from deciduous (*t* = 5.6; *P* = 0.003) and brevideciduous species (*t* = 3.5; *P* = 0.008). In the categories formed by section and leaf habit we found significant differences in three traits: vessel diameter (*F* = 4.95; *P* = 0.01), wood density (*F* = 3.49; *P* = 0.01) and relative hydraulic conductivity (*F* = 6.67; *P* = 0.001). *Post hoc* tests showed that vessel diameter in white brevideciduous is significantly wider than red deciduous oaks. For wood density, white deciduous oaks are denser than red brevideciduous oaks. Finally, white deciduous oaks possess higher relative hydraulic conductivity than red deciduous and brevideciduous oaks, and red brevideciduous oaks possess lower relative hydraulic conductivity than white brevideciduous oaks (Fig. 5).

Lastly, the wood traits tested in this study showed low phylogenetic signals based on Blomberg’s *K* (*K* < 0.35; **see****Supporting Information—Table S5**). Only three traits showed significant values [wood density (*K* = 0.23; *P* = 0.014), vessel diameter (*K* = 0.31; *P* = 0.015); and relative hydraulic conductivity (*K* = 0.35; *P* = 0.004)].

## Discussion

### Covariation in oak wood traits

Oak species show a wide range of morphological and anatomical responses to environmental conditions (Cavender-Bares *et al.* 2018; Lobo *et al.* 2018; Skelton *et al.* 2021). In the present study, the results of Pearson’s and PICs correlations analyses were generally congruent. The correlations among PICs showed lower correlation coefficients or even became insignificant, as also found by other studies (Carvalho *et al.* 2006; Jacobsen *et al.* 2007; Fu *et al.* 2012). These results indicated that correlations might be decreased because they are based on labile traits. It is important to consider that evolutionary correlations between these labile traits tend to be much lower than those between conservative traits (Carvalho *et al.* 2006). However, we have been able to identify some relationships in anatomical traits previously mentioned in literature as the negative relationship between vessel diameter and frequency (Table 2; Fig. 3), and both varied inversely with wood density. In the oak species analysed wide vessels in lower frequency lead to lower wood densities. Wood density was also negative correlated with fibre lumen, showing that the increase in wood density was driven by a decrease in fibres diameter and lumen, which would mean that having more fibres with a smaller diameter makes them heavier. This is consistent with previous works (Jacobsen *et al.* 2005; Martínez-Cabrera *et al.* 2009) that showed that denser woods had more fibre cells per unit area than lighter woods (Martínez-Cabrera *et al.* 2009; Ziemińska *et al.* 2015). The variation in wood density across species has been explained by changes in the fibre wall and lumen fractions (Zanne *et al.* 2010; Ziemińska *et al.* 2013), and as a response to environmental conditions as temperature or precipitation (Swenson and Enquist 2007; Martínez-Cabrera *et al.* 2009) although not in all studies. In lowlands, the high temperatures (30 °C mean annual temperature; 36 °C maximum temperature of the driest quarter) in combination with periodic water deficits lead to narrower vessels immersed in a matrix of high-density wood due to the higher proportion of xylem occupied by smaller fibres, which increase the resistance to implosion (Jacobsen *et al.* 2007; Fortunel *et al.* 2014). On the contrary, at high altitudes with lower temperatures (18 °C mean annual temperature; 21 °C maximum temperature of the driest quarter) and higher amount of rainfall throw the year, oaks invest in widest vessels to conduct water with great efficiency and increasing conductivity in combination with lower wood density (Hacke *et al.* 2006; Fan *et al.* 2011).

Plant height is an important trait related with water balance, carbohydrate transport and light interception (H. Liu *et al.* 2019). The positive relationship among vessel diameter and tree height has previously mentioned in other studies (Preston *et al.* 2006; Martínez-Cabrera *et al.* 2009; Cosme *et al.* 2017; Olson *et al*. 2018) among others. This relationship has been suggested as a coordinated evolutionary change on water transport to maximize the water flow (Preston *et al.* 2006). However, wider conducts are more vulnerable due to the biophysical constraints on water transport to canopies, the risk of cavitation increase (Niklas and Spatz 2004; H. Liu *et al.* 2019). Therefore, trees should produce conduits no wider than those permitted by embolism risk given microsite conditions (such as water availability, temperature, rooting depth and soil type) and height ecological strategy (Olson *et al*. 2018).

### Climate trait relationship

In this study, wood traits reflect a coordinated strategy among storing and transporting water and prevent embolism by seasonal environmental conditions, where oak species in drier environments increase wood density by reducing fibre lumen while increasing storage capacity and adjust water flow reducing their vessel size. Generally, vessel diameter increases, and vessel frequency decreases with an increase in precipitation and water availability across different habitats (Carlquist 1988; Machado *et al.* 2007). Our climate analyses showed that AI and seasonal regimens of temperature and precipitation play an essential role in shaping wood anatomical traits and variables. Global analyses found that taller woody species occur in biomes with higher water availability, higher xylem hydraulic conductivity and are more vulnerable to xylem embolism (Z. Liu *et al.* 2019). Our results with the pgls found that wood density, relative hydraulic conductivity and tree height were significantly driven by the AI. Oak species in dry areas with the lowest aridity value (AI = 0.78) in lowlands possess high wood densities and lower relative hydraulic conductivity, and in higher altitudes with higher aridity values (AI = 1.78) humid places possess taller oaks with higher relative hydraulic conductivity and lower wood density. Seasonal patterns in temperature and precipitation influence ecological process as seedling growth, productivity and phenology among others, all of which have an impact on the survival and establishment of plants in mountainous areas (Tang and Fang 2006; Salamon-Albert *et al.* 2017). Seasonal precipitation regimen is extremely relevant such as precipitation of warmest quarter because it determines the highest level of drought stress, and plants must avoid or tolerate the drought. Previous findings that linked increasing wood mechanical strength with increasing cavitation resistance have found a mechanical reinforcement of vessels by the neighbouring fibre matrix (Hacke *et al.* 2001; Jacobsen *et al.* 2005, 2007) or with small vessels (<15 μm) or tracheids and vasicentric tracheids plus the parenchyma could also play an auxiliary role during stress, such as drought (Carlquist 1985; Ziemińska *et al.* 2015). The occurrence of vasicentric tracheids appears to be an anatomical structure to survive for oaks during drought conditions in different environments around the world (Carlquist 1985; Sousa *et al.* 2009; Gupta and Gupta 2020; Percolla *et al*. 2021), and in this study, but more research on this structure is needed (Pan and Tyree 2019; Fontes and Cavender-Bares 2020).

### Plasticity in oaks

Plants exhibit strong plasticity when are exposed to environmental changes (Valladares *et al.* 2006). In this study, we found that oak species distributed along contrasting environmental sites showed higher plasticity, where higher environmental distance showed higher RDPI values. In general, we found low RDPI values and red oaks possess higher RDPI values than white oaks although the white oaks have a wider distribution in the study area; and at the leaf habit category, deciduous oak species possess higher RDPI values. Several studies have shown low plasticity across populations in other species (Scholz *et al.* 2014; Salazar *et al.* 2019) or in species with no variation along an aridity gradient (e.g. *Q. petraea*) in some vessel traits, suggesting that other anatomical features (e.g. parenchyma or vasicentric tracheids) regulate drought tolerance (Sousa *et al.* 2009; Lobo *et al.* 2018). This implies that there is still great anatomical variation in oak wood cells that can lead to mechanical stability (Ziemińska *et al.* 2015; Morris *et al.* 2018; Percolla *et al*. 2021). In addition, we need to complement the study of intraspecific functional trait covariance in all the distribution range for each species in wood anatomical structures to understand the adaptive value of trait combinations for predicting species responses to changing environmental conditions. Species with lowest RDPI could have limited variability in the analysed traits, which may reduce their ability to respond to changing climates.

### Co-occurrence among oaks

The probabilistic model that we use will classify as ‘random’ two widespread species that occur in a high proportion of the sampling sites (Veech 2013). In our results the high proportion of random co-occurrence species pairs suggests that the two oaks species are distributed independently of one another and that the small fraction of positive species pairs could be attributed to shared environmental responses (Royan *et al.* 2016). On the other hand, the positive co-occurrence patterns can be interpreted as a niche partitioning process in which different resources acquisition and conservation strategies allow them to survive (Veech 2013). The positive co-occurrence patterns can be explained by shared environmental responses, suggesting that environmental filtering is an important mechanism that operates to structure oak assemblages (Cavender-Bares *et al.* 2004; Fallon and Cavender-Bares 2018; Arenas-Navarro *et al.* 2020b). Previous studies have suggested differentiation in hydraulic traits among phenological groups, finding differences among co-occurring tree species related to leaf and stem structural traits with different leaf habits (Markesteijn *et al.* 2011; Scholz *et al.* 2014; Santini *et al*. 2016; Zhang *et al.* 2017). Eight of the nine positive associations detected are composed of white and red oaks; this matches the expectation that more closely related species will show greater habitat separation, and oak species from different sections will tend to coexist (Cavender-Bares *et al.* 2004; Fallon and Cavender-Bares 2018; Teshera-Levye *et al.* 2020). Also, four positive species pairs showed different leaf habit strategy, which reflect a different drought-avoidant strategy. This result suggests that oaks species were consistent with phylogenetic relatedness as a driver for community structure and functional diversification as in other study sites (Cavender-Bares *et al.* 2004; Fallon and Cavender-Bares 2018; Teshera-Levye *et al.* 2020).

### The influence of leaf habit

The drought-avoidant strategies of water use are coordinated between leaf and wood tissues (Díaz and Cabido 2001; Méndez-Alonzo *et al.* 2012; Reich 2014). Our results showed that the main differences among wood anatomical traits and variables are leading by leaf habit. Leaf habit has been associated with traits related to the leaf economic spectrum (LES) (Wright *et al.* 2004; Fu *et al.* 2012). The LES is the trade-off between high resource acquisition and resource conservation strategies (Wright *et al.* 2004; Reich 2014); these leaf strategies have benefits in the face of alternating stress. Leaf habit strategies show a great variation; for example, evergreen species may have long leaf longevity or short leaf longevity but maintain foliage life for extended periods accompanied by constant leaf replacement (Brodribb and Holbrook 2005; Fu *et al.* 2012; Ribeiro *et al.* 2021). In contrast, deciduous species show high variability in leaf longevity reflecting local climatic seasonality (Singh and Kushwaha 2005; Kikuzawa and Lechowicz 2011; Ribeiro *et al.* 2021). We found significant differences among the three leaf habit categories in vessel diameter; the widest vessels (> 230 µm) were observed in tall evergreen oaks located in humid places, in contrast to the narrow vessels (150–200 µm) in deciduous oaks in driest places. Also, evergreen oaks have a significantly wider fibre lumen than deciduous. Contrary, we did not find significant differences among deciduous and brevideciduous oaks. This could be due that the brevideciduous category is a subcategory that reflects a general status of the canopy (Brodribb and Holbrook 2005; Kikuzawa and Lechowicz 2011); likewise, it has been shown that leaf physiological characteristics affected leaf phenology when it was described as canopy foliage duration better than when described as leaf longevity (González-Rebeles *et al.* 2021). In deciduous species, leaves are discarded when leaf maintenance costs exceed gains, but prior to the leaf shedding, water and nutrients are translocated from leaves to other plant organs (Singh and Kushwaha 2005).

In environments with low water availability, the species with resistance to xylem cavitation via wood traits closely coordinated with leaf traits are favoured (Ribeiro *et al.* 2021). This coordination includes variation in xylem wood porosity, vessel diameter and pit length, resistance and durability of the pit membrane, and wood density (Hacke *et al.* 2001; Méndez-Alonzo *et al.* 2012). In India’s seasonally dry tropical forest, deciduous species responded similarly to rainfall seasonality, but species with the lowest deciduousness had the highest wood density (Kushwaha *et al.* 2010). Showing that wood density can decrease in an inverse proportion with the duration of deciduousness of species in some environments (Kushwaha *et al.* 2010; Chaturvedi *et al.* 2021).

In the groups formed by section and leaf habit, we found differences in vessel diameter, wood density and relative hydraulic conductivity. Relative hydraulic conductivity reflects the variation in efficiency and susceptibility caused by the vessel diameter despite concurrent increases in the vulnerability to xylem cavitation (Carlquist 1988; Gutiérrez *et al.* 2009). In some cases, xylem cavitation is avoided by the partial or total displacement of leaves, but it can also be achieved by decreasing the efficiency in xylem conductance under negative pressure by investing in vessels with narrow diameters and thick and rigid cell walls (Hacke *et al.* 2001; Méndez-Alonzo *et al.* 2012). In our study, RC acts as a determinant factor to define the groups formed by section and leaf habit. White brevideciduous oaks have higher RC and red deciduous oaks have the lower RC. Deciduous and brevideciduous oaks species in lowlands shed their leaves at the beginning of the dry season in spring, the species with narrower vessels and high frequency are safer raising the potential for hydraulic conductivity (Jacobsen *et al.* 2007; Fortunel *et al.* 2014). However, the deciduous and brevideciduous oaks species that exhibit a wider diameter will be operating under safer hydraulic limits with anatomical structures to protect of embolism as vasicentric tracheids or the presence of tyloses among others (Pérez de Lis *et al.* 2018; Percolla *et al*. 2021).

### Differences among sections and phylogenetic signal

The phylogenetic signal describes a tendency for evolutionarily related species to resemble each other; with no implications as to the mechanisms that might cause such resemblance (Blomberg *et al.* 2003), where the lack of phylogenetic signal implies evolutionary lability (Silvertown *et al.* 2006b). In a phylogeny, heritable traits that vary freely among the terminals of the tree are likely to be evolutionary labile (labile traits) and traits that vary little among terminals on the same tree indicate that their evolution is more conservative (conservative traits) (Silvertown *et al.* 2006a).

Vessel diameter and wood density are important wood characteristics that are considered traits with phylogenetic conservatism in some genera and families (Fisher *et al.* 2007; Scholz *et al.* 2014). Likewise, studies with oaks have found that vessel diameter is a conserved trait (Cavender-Bares *et al.* 2004; Robert *et al.* 2017). Also, recent analysis in four clades of North American oaks found a phylogenetic signal for stem P_50_ (*K* = 0.63; xylem water potential value at which 50 % loss of hydraulic conductance occurs; Skelton *et al.* 2021), indicating that close relatives tend to show phylogenetic conservatism. However, we did not find that vessel diameter and wood density are conserved traits in the oak species analyse.

Oaks radiated in North America starting ca. 35 Ma when the temperate forest biome was moved southward to Mexico due to climatic changes and tropical taxa loss (Manos and Stanford 2001; Hipp *et al.* 2018). *Quercus* and *Lobatae* radiated in sympatry and colonized western and eastern North America in parallel and subsequently radiated southward along the Mexican highlands diversifying in more than 154 oak species in Mexico (Hipp *et al.* 2018). In the case of Mexican oaks, it has been pointed out that the high lability of the traits along the humidity gradients in the newly available habitats allowed them to be extraordinarily diversified (Hipp *et al.* 2018). It has been proposed that when phylogenetic conservatism is strong, species may experience difficulties to colonize and to adapt to new environments (Losos 2008), while rapidly evolving traits may fuel the spread into new zones, and clades can experience greater diversification success (Holt 1990; Martínez-Cabrera *et al.* 2017). This may explain why in the hotspot studied low phylogenetic signal was found.

Analysing the variation in wood anatomical traits and variables can help us to understand the adaptations in oak species by interpreting the variation of wood structures across environmental gradients. This study found that high temperatures in combination with periodic water deficits lead to narrower vessels, high-density wood occupied by smaller fibres. On the contrary, on humid sites tall oaks invest in widest vessels to conduct water with great efficiency and increasing conductivity in combination with lower wood density. In the SMJal the oaks species showed an adaptive response of wood traits to climate, but there is also evidence of a low phylogenetic signal. Co-occurrence of oak species with different leaf habits and phylogenetic trajectories may promote complementary resource acquisition. The combination of plasticity and lability in wood traits among Mexican oaks gives a particular trait configuration for the water-use strategy along environmental gradients.

## Supplementary Information

The following additional information is available in the online version of this article—

Table S1. *Quercus* (oaks) species analysed in this study with their phylogenetic section and leaf habit category.

Table S2. Variable scores of principal components analysis (PCA).

Table S3. Relationship between anatomical and hydraulic traits of oak species.

Table S4. Phyloanova results.

Table S5. Blomberg’s *K* values.

Figure S1. Maps of the study area and sampling plots.

Figure S2. Relative distance plasticity index (RDPI) values for each oak species.

Figure S3. Multiple regression on distance matrices (MRM) by section among relative distance plasticity index (RDPI) values and environmental distance and geographic distance (km).

plab066_suppl_Supplementary_Material

## Data Availability

Data traits used for analysis in this publication can be found in **Supporting Information—Table S1**.

## Notes

**Form & Function. Chief Editor**: Kate McCulloh
