# Supplementary material for: The role of wood anatomical traits in the coexistence of oak species along an environmental gradient
Source: AoB Plants. 2021 Oct 18;13(6):plab066. doi: 10.1093/aobpla/plab066 (PMC8633429; doi:10.1093/aobpla/plab066)

Article title: **The role of wood anatomical traits in the coexistence of oak species along an environmental gradient**

Authors: Maribel Arenas-Navarro, Ken Oyama, Felipe García-Oliva, Andrés Torres-Miranda, Enrique G. de la Riva, Teresa Terrazas

Table S1. *Quercus* (oaks) species analyzed in this study with their phylogenetic section and leaf habit category. Means of wood anatomical traits by species, coefficient of variation is presented in parentheses. Acronyms: Sec (Section), Lob (*Lobatae*), Que (*Quercus*), LH (Leaf habit), dec (deciduous), bre (brevideciduous), eve (evergreen), VD (Vessel Diameter; µm), VF (Vessel Frequency; /mm²), F_D (Fiber total diameter; µm), F_L (Fiber lumen; µm), F_W (Fiber wall; µm), WD (wood density; g/cm3), H (tree height; m) and DBH (Diameter breast height; cm).

Species	Sec.	LH	VD	VF	F_D	F_L	F_W	WD	H	DBH
Q. aristata Hook. & Arn.	*Lob*	dec	169.51 (0.8)	7 (0.2)	16.5 (.15)	4.7 (.4)	5.8 (.14)	0.72 (0.13)	7.21 (0.38)	19.90 (0.37)
Q. calophylla Schltdl. & Cham.	*Lob*	bre	199.7 (0.05)	5 (0.26)	23.5 (0.15)	7.3 (.2)	7.2 (.12)	0.61 (0.09)	9.50 (0.41)	39.90 (0.3)
Q. castanea Née	*Lob*	bre	170.8 (0.09)	7 (0.32)	19.1 (0.05)	7.5 (0.08)	5.8 (0.08)	0.59 (0.09)	14.25 (0.42)	41.84 (0.28)
Q. crassifolia Bonpl.	*Lob*	bre	183.9 (0.05)	6 (0.17)	16.2 (0.10)	6.4 (0.2)	4.8 (0.11)	0.56 (0.12)	7.33 (0.36)	24.73 (0.38)
Q. cualensis L.M. González	*Lob*	bre	191.4 (0.10)	6 (0.26)	19 (0.07)	7.7 (0.16)	5.6 (0.12)	0.71 (0.1)	8.5 (0.39)	21.75 (0.17)
Q. eduardii Trel.	*Lob*	bre	178.1 (0.16)	6 (0.30)	19.1 (0.09)	5.6 (0.15)	6.7 (0.15)	0.58 (0.1)	11.78 (0.5)	25.89 (0.18)
Q. elliptica Née	*Lob*	dec	180.8 (0.19)	4 (0.22)	19.2 (0.12)	5.7 (0.28)	7.4 (0.21)	0.67 (0.09)	8.40 (0.35)	26.60 (0.30)
Q. glaucescens Bonpl.	*Que*	bre	219.6 (0.16)	6 (0.17)	20.3 (0.11)	5.3 (0.27)	7.5 (0.12)	0.71 (0.09)	9.62 (0.36)	27.05 (0.29)
Q. iltisii L.M. González	*Lob*	bre	201.3 (0.10)	5 (0.17)	16.1 (0.21)	4.1 (0.32)	6 (0.2)	0.68 (0.08)	11.50 (0.20)	32.08 (0.39)
Q. jonesii Trel.	*Lob*	dec	156.1 (0.15)	8 (0.3)	21.0 (0.13)	7.2 (0.22)	7.3 (0.16)	0.60 (0.09)	6 (0.23)	19.89 (0.37)
Q. laeta Liebm.	*Que*	dec	196.5 (0.10)	7 (0.20)	18.5 (0.08)	5.4 (0.35)	6.3 (0.15)	0.66 (0.12)	9.73 (0.32)	37.52 (0.25)
Q. laurina Bonpl.	*Lob*	bre	202.0 (0.11)	5 (0.20)	20.3 (0.16)	7.9 (0.25)	6.4 (0.19)	0.57 (0.16)	21.80 (0.14)	64.13 (0.18)
Q. liebmannii Oerst. ex Trel.	*Que*	dec	201.0 (0.04)	5 (0.15)	18.5 (.08)	5.3 (0.28)	6.7 (0.11)	0.74 (0.12)	9.60 (0.14)	29.28 (0.32)
Q. magnoliifolia Née	*Que*	dec	188.7 (0.05)	6 (0.21)	17.6 (0.17)	4.8 (0.27)	6.3 (0.17)	0.70 (0.1)	6 (0.26)	24.77 (0.26)
Q. martinezii C.H. Mull.	*Que*	eve	263.7 (0.07)	5 (0.2)	21.6 (.02)	7 (0.24)	7.2 (0.11)	0.61 (0.03)	22.8 (0.05)	62.07 (0.34)
Q. mexiae L.M. González	*Lob*	dec	171.8 (0.10)	6 (0.30)	21.9 (10)	6.6 (0.28)	7.4 (0.13)	0.70 (0.14)	5.5 (0.25)	18.14 (0.09)
Q. obtusata Bonpl.	*Que*	bre	196.6 (0.09)	6 (0.11)	22.7 (.11)	7.2 (0.09)	6.8 (0.11)	0.65 (0.08)	11.3 (0.36)	32.01 (0.28)
Q. resinosa Liebm.	*Que*	dec	190.9 (0.03)	7 (0.14)	22.1 (.08)	5.2 (0.18)	8.3 (0.09)	0.70 (0.10)	8.4 (0.17)	27.90 (0.57)
Q. scytophylla Liebm.	*Lob*	bre	177.2 (0.12)	6 (0.12)	18.4 (.04)	5.3 (0.18)	6.5 (0.07)	0.68 (0.11)	9.50 (0.25)	32.19 (0.34)
Q. tuitensis L.M. González	*Lob*	dec	190 (0.03)	6 (0.23)	15.7 (.13)	5.2 (0.27)	5.2 (0.16)	0.73 (0.11)	6.82 (0.32)	23.28 (0.14)
Q. uxoris McVaugh	*Lob*	eve	218.4 (0.12)	5 (0.17)	19.2 (.07)	8 (0.15)	7.2 (0.18)	0.62 (0.02)	20.4 (0.06)	59.96 (0.35)

Table S2. Variable scores of PCA. Bold numbers are the highest contribution of each component.

	Traits			PICs		
Trait	Dim.1	Dim.2	Dim.3	Dim.1.	Dim.2	Dim.3
VD	**-0.9652**	0.06	-0.21	**0.89**	-0.30	0.18
VF	0.54	-0.51	-0.05	-0.31	**0.82**	0.44
F_D	-0.24	**-0.84**	-0.01	0.40	0.57	**-0.66**
F_L	-0.52	-0.21	0.68	0.65	0.25	-0.36
F_W	0.03	-0.68	-0.38	0.25	0.62	-0.62
WD	-0.01	0.43	**-0.71**	-0.59	-0.05	-0.13
VI	-0.83	0.35	0.01	0.64	-0.69	-0.26
RC	-0.88	-0.17	0.31	0.84	0.12	0.37
F	-0.69	-0.32	-0.33	0.55	0.50	0.62
H	-0.83	0.03	0.37	0.86	-0.003	0.07

Table S3. Relationship between anatomical and hydraulic traits of oak species. Phylogenetic generalized least squares (pgls) and ordinary least squares (ols). Significance levels are shown.

**Trait**	**Environmental variable**	**PGLS**		**OLS**	
		R2 adj	*P*	R2 adj	*P*
VD	Aridity index	0.06	0.14	-0.03	0.58
VF	Precipitation of Wettest Quarter	0.17	0.04	0.15	0.05
F_D	Precipitation of Wettest Quarter	0.01	0.27	-0.01	0.4
F_L	Mean Temperature of Driest Quarter	0.63	0.004	0.63	0.00005
F_W	Mean Temperature of Driest Quarter	-0.008	0.36	0.28	0.01
WD	Aridity index	0.37	0.003	0.35	0.004
VI	Precipitation of Warmest Quarter	0.2	0.02	0.2	0.029
RC	Aridity index	0.18	0.03	-0.02	0.44
F	Precipitation of Wettest Quarter	0.46	0.0005	0.02	0.246
H	Aridity index	0.24	0.01	0.21	0.03

Table S4. Phyloanova results. Phyloanova among *Quercus* and *Lobatae* sections; among leaf habit (deciduous, brevideciduous and evergreen) and among section and leaf habit groups. Significant correlations are shown in bold.

	**Section**		**Leaf habit**		**Section/Leaf habit**	
	F	*P phy*	*F*	*P phy*	*F*	*P phy*
**VD**	4.18	0.84	12.02	**0.001**	**4.95**	**0.01**
**VF**	0.22	0.99	0.87	0.66	0.08	0.96
**F_D**	1.37	0.93	0.06	0.98	0.68	0.57
**F_W**	9.84	0.62	0.86	0.64	0.76	0.53
**F_L**	2.3	0.88	4.8	**0.04**	1.15	0.63
**WD**	4.04	0.82	2.34	0.17	**3.49**	**0.04**
**VI**	0.69	0.96	4.58	**0.03**	0.495	0.693
**RC**	6.65	0.75	7.23	**0.008**	**9.679**	**0.001**
**F**	4.96	0.78	4.47	**0.04**	2.98	0.07
**S**	2.008	0.91	8.63	**0.001**	0.894	0.473
**H**	1.36	0.93	17.23	**0.001**	2.56	0.104

Table S5. Blomberg's *K* values. *P* values for traits with significant phylogenetic signal are highlighted in bold. Tests are based on the method of Blomberg, Garland & Ives (2003) using 999 permutations of the null model as implemented in the R package 'picante'.

	K	***P***
VD	**0.31**	**0.015**
VF	0.10	0.608
F_D	0.08	0.817
F_L	0.19	0.199
F_W	0.21	0.246
WD	**0.23**	**0.014**
VI	0.17	0.159
RC	**0.35**	**0.004**
F	0.17	0.221
S	0.21	0.073
H	0.28	0.058

Figures

Figure S1. Maps of the study area and sampling plots.

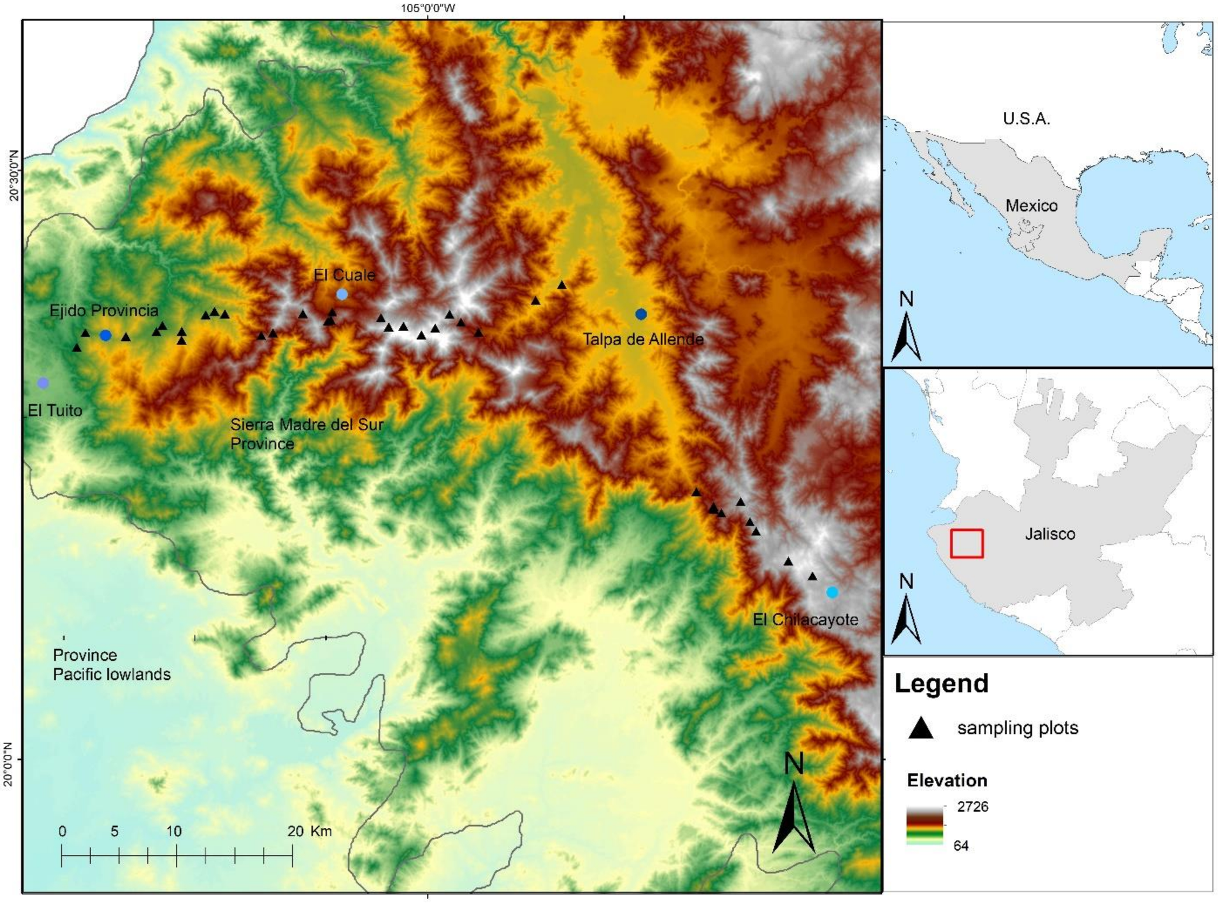

Figure S2. Relative distance plasticity index (RDPI) values for each oak specie. RDPI ranges from 0 (no plasticity) to 1 (maximal plasticity). a) Wood anatomical traits; b) Anatomical variables.

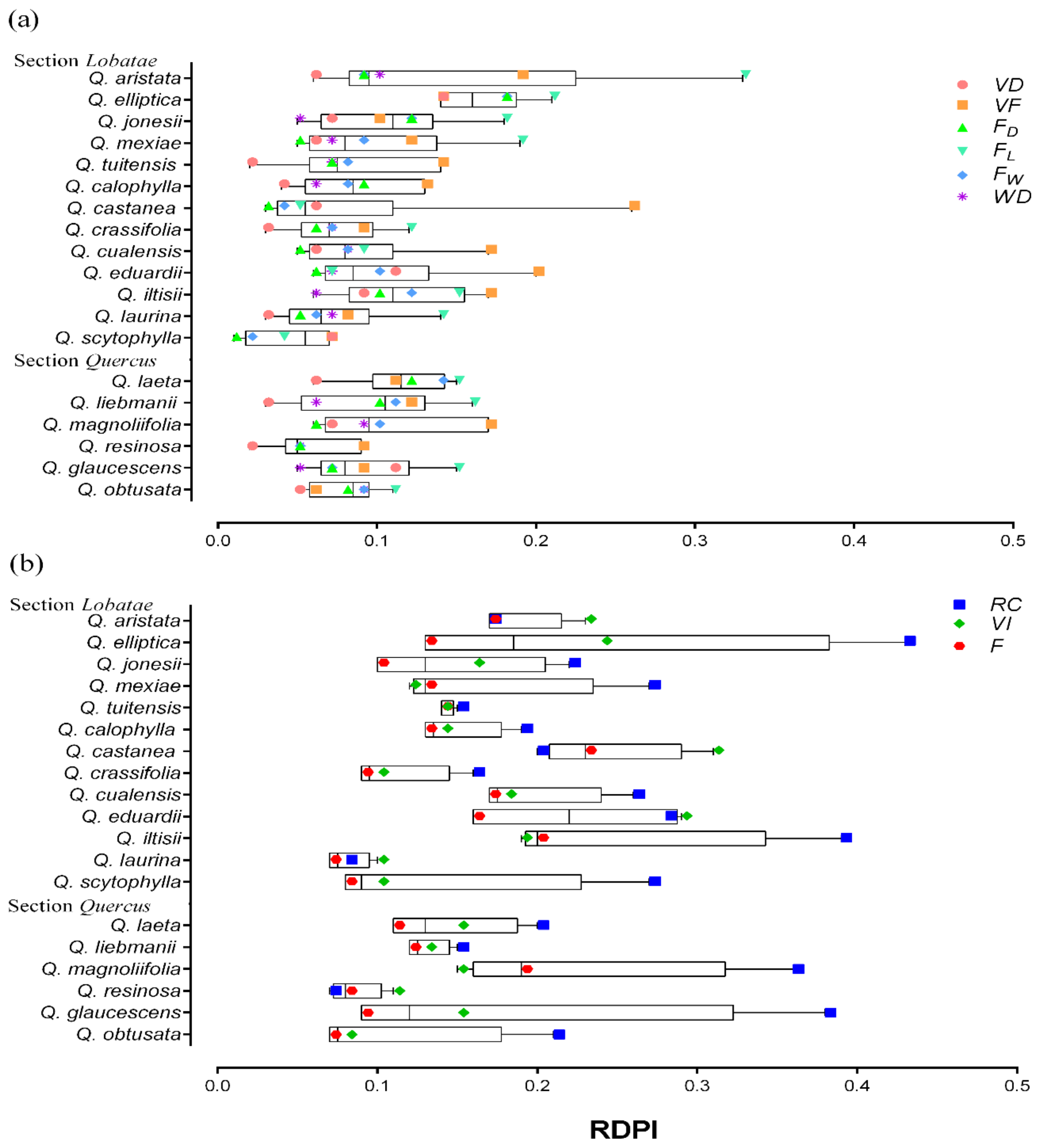

Figure S3. Multiple regression on distance matrices (MRM) by section among relative distance plasticity index (RDPI) values and environmental distance and geographic distance (km). Red oaks are a-d panels. White oaks are e-h panels. Significance levels are shown. NS=No significative; *P* < 0.05, *; *P* < 0.001, **. The dotted lines are the ordinate to the origin at zero for each model.

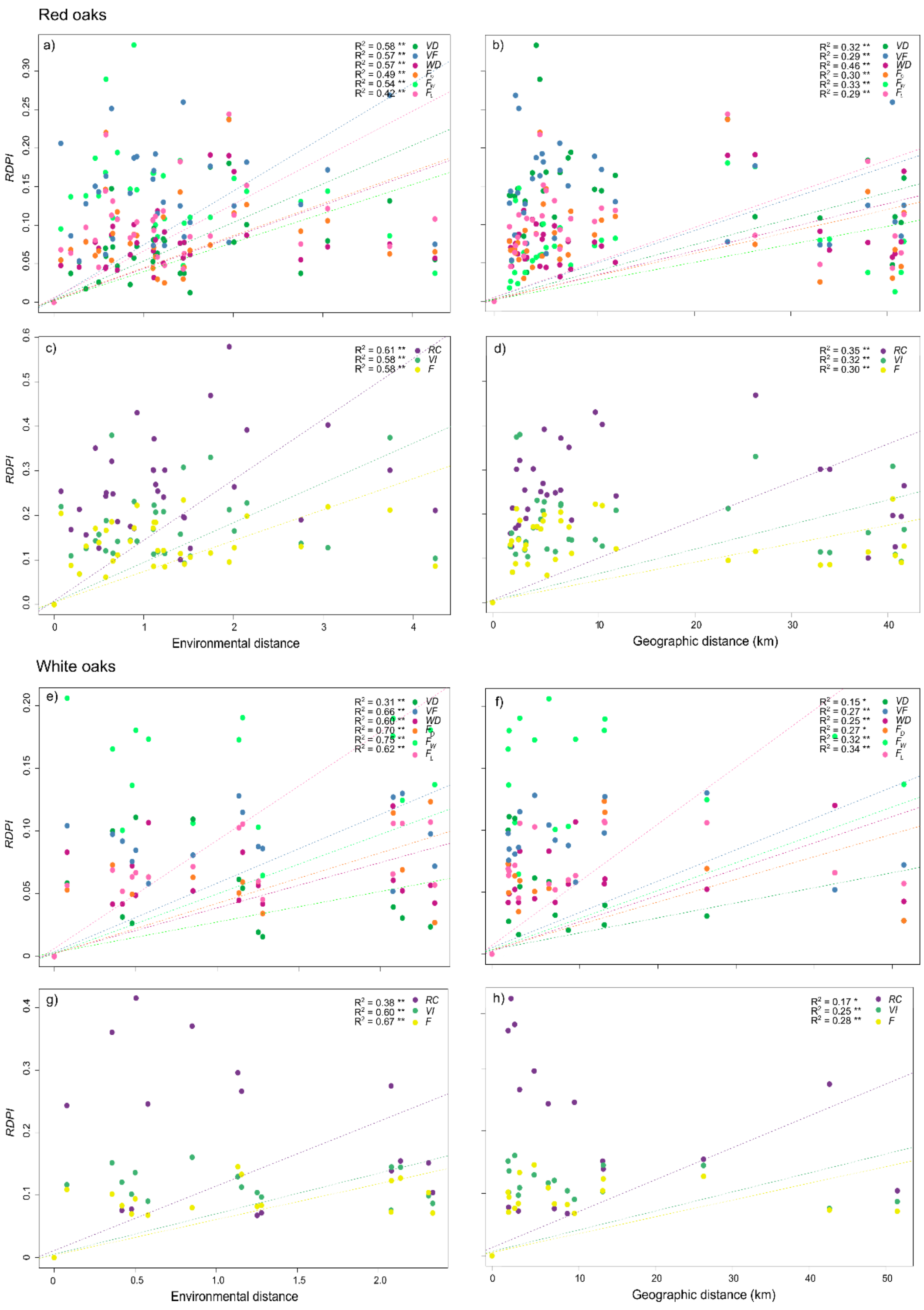

Supplement: plab066_suppl_Supplementary_Material [file plab066_suppl_supplementary_material.pdf]
